# PENSION AND SUBJECTIVE WELL-BEING AMONG OLDER ADULTS IN URBAN CHINA: THE ROLE OF SOCIAL PARTICIPATION

**DOI:** 10.1093/geroni/igz038.3309

**Published:** 2019-11-08

**Authors:** haojun jiang, Iris Chi

**Affiliations:** 1 nanjing agricultural university, nanjing, China; 2 University of Southern California, Los Angeles, California, United States

## Abstract

This study highlighted the relationship and interaction mechanism among pension, social participation and subjective well-being of older adults in urban China, which provided cross-cultural evidence for theories, and had significant implications for social security policy. It examined the relationship between pension and subjective well-being (i.e., life satisfaction, depression) among older adults in urban China. It also assessed the mediating and moderating effects of social participation (i.e., three types of activity participations including labor activity participation, political activity participation, voluntary activity participation; and the variation of activity participation) in the linkage between pension and subjective well-being. The data came from the 2014 China Longitudinal Aging Social Survey (CLASS), a national, large-scale survey of a representative community aging sample (60 years and older) in urban China (N=6907). The study used hierarchical regression analysis and structural equation modeling methods. The results showed that pension could improve the subjective well-being of the older adults, specifically, pension enhances life satisfaction and reduces the depression of older adults. Both of three types of activity participation and the variation of activity participation were a significant moderator in the relationship between pension and subjective well-being. Besides, both labor activity participation and variation of activity participation were also a partial mediator in the relationship between pension and subjective well-being.

